# Experimental evolution of plant rhizobacteria reveals emerging adaptive mutations

**DOI:** 10.1128/mbio.01023-25

**Published:** 2025-07-14

**Authors:** Jiahui Li, Yingying Zhang, Wenjun Jiang, Li-Qun Zhang

**Affiliations:** 1 Department of Plant Pathology and Ministry of Agriculture and Rural Affairs Key Laboratory of Pest Monitoring and Green Management, College of Plant Protection, China Agricultural University 539443 , Beijing, China; Indiana University Bloomington, Bloomington, Indiana, USA; University of Pittsburgh, Pittsburgh, Pennsylvania, USA

**Keywords:** experimental evolution (EE), *Pseudomonas bijieensis *2P24, single-nucleotide polymorphism (SNP), wheat rhizosphere, adaptive colonization, bacterial flagella

## Abstract

The colonization capability of plant growth-promoting rhizobacteria (PGPRs) in the rhizosphere is essential for their beneficial effects on plant growth, yet the evolutionary processes and adaptive potential of PGPRs in this environment remain underexplored. Here, we established an experimental evolution system for the wheat rhizosphere using *Pseudomonas bijieensis* 2P24 (former name: *Pseudomonas fluorescens* 2P24) as a model, simulating its natural evolution and tracking its genomic alterations to uncover adaptive mutations. Whole-genome sequencing and single-nucleotide polymorphism analysis revealed the accumulation of mutations in the flagella-associated gene *fleN* during evolution, reaching 49% frequency in the final communities. These mutations increased the number of bacterial flagella, which in turn enhanced motility and colonization capacity in the wheat rhizosphere compared to the ancestral strain. This study represents the first investigation of PGPR adaptive evolution in the wheat rhizosphere, providing a system for studying plant-associated bacteria and insights into selecting and engineering PGPR strains for agricultural applications.

**IMPORTANCE:**

Root colonization ability results from the long-term evolutionary adaptation of certain bacteria to the plant rhizosphere, involving extensive bacterial genetic resources. Understanding colonization mechanisms is crucial for fully exploiting the potential of PGPRs. *Pseudomonas* spp. are important biocontrol agents for plant diseases, with strong affinity for plant roots and large populations in the rhizosphere, making them key models for studying PGPR colonization mechanisms. Most studies on *Pseudomonas* colonization rely on molecular genetics and omics approaches, which reveal many bacterial traits and mechanisms involved in rhizosphere colonization but are limited in detecting continuous genomic changes and subtle nucleotide variations. In this study, we established an experimental evolution system using *Pseudomonas bijieensis* 2P24 in the wheat rhizosphere to simulate bacterial evolution in the plant rhizosphere. We observed that bacteria enhance colonization ability by fine-tuning the flagellar number, revealing a novel adaptive mutation in plant rhizobacteria.

## INTRODUCTION

The colonization of the rhizosphere by plant growth-promoting rhizobacteria (PGPRs) not only is a fundamental step in the effective control of soil-borne diseases but also represents a complex biological and ecological process. This process encompasses the initial sensing of rhizosphere signals by indigenous bacteria, their subsequent chemotactic movement and motility, the identification and selection of specific colonization sites, the overcoming of host immune responses, competition with other environmental microbes, and the eventual formation of complex bacterial communities in the rhizosphere (1, 2). Unraveling the adaptive potential of these biocontrol bacteria is essential for informing the selection and development of potent biocontrol strains designed for agricultural applications.

The colonization of the rhizosphere by bacteria is inherently an adaptive evolutionary process. Current molecular genetics and omics-based research on *Pseudomonas* colonization have identified colonization-associated genes through functional gain or loss studies and have analyzed microbial species, functional genes, and their expression profiles under rhizospheric and non-rhizospheric conditions to identify factors influencing colonization (3–9). While these approaches have successfully elucidated numerous bacterial traits and mechanisms involved in rhizosphere colonization, they have limitations, particularly in capturing the dynamic and continuous changes in bacteria as they adapt to the rhizosphere.

The study of genomic modifications in bacteria during adaptive evolution through experimental evolution provides a powerful strategy to uncover the adaptive potential of bacteria (10). Throughout this process, bacteria develop enhanced adaptability, driven by competitive differences under experimental environmental pressures (11, 12). Whole-genome sequencing techniques enable the detection of all genomic modifications in evolved isolates compared to the ancestral strain, offering insights into the genetic basis of bacterial adaptive evolution (13, 14). By integrating experimental evolution with comparative genomics, it is possible to track the genetic evolution of bacteria in real time, correlating observed phenotypic changes with genetic variations and thus gaining a comprehensive understanding of the trajectory of bacterial adaptive evolution (15). While experimental evolution methods are primarily used to study bacterial and microbial evolution in controlled environments, there is limited knowledge about the direct adaptive evolution of microorganisms on plants (15). Our research investigates the evolutionary processes of *Pseudomonas* as it adapts to the rhizosphere of crops, aiming to elucidate the mechanisms by which PGPRs function in the plant rhizosphere.

*Pseudomonas bijieensis* 2P24 (former name: *Pseudomonas fluorescens* 2P24), isolated from soil naturally suppressive to wheat take-all disease, has demonstrated effective biocontrol against various soil-borne diseases, including tomato wilt (16). In this study, we conducted an evolutionary experiment on the 2P24 strain over 297 generations in the wheat rhizosphere, establishing four independent evolved lines and subjecting 16 evolved populations to metagenomic sequencing. After the experimental evolution, we performed phenotypic analysis of the most prevalent flagellar gene *fleN* mutants in these populations to assess their adaptive gains in the wheat rhizosphere. The results indicated that 2P24 rapidly differentiated into genotypes with enhanced colonization capacities in the wheat rhizosphere, positively impacting its adaptation and offering a competitive advantage over the ancestral genotype.

Bacterial flagella play a crucial role in plant-microbe interactions (17). In the rhizosphere, bacteria utilize flagellar rotation to colonize roots and form biofilms (18, 19). However, the impact of bacterial flagellar number on root colonization capacity remains poorly understood. In this experiment, we observed an increased mutation frequency of the key flagellar number-related gene, *fleN*, across all four independently evolved populations (20, 21). Point mutations in the *fleN* gene fine-tuned the bacterial flagellar number, with a significant correlation between an optimal increase in 2P24 flagellar number and enhanced colonization. Characterization of multiple flagella-related phenotypes in mutant strains provided new insights into how bacterial genomic modifications enhance adaptability in the plant rhizosphere, contributing to our understanding of the role of bacterial genomic modifications in rhizosphere evolution.

## RESULTS

### Adaptive experimental evolution of *Pseudomonas bijieensis* 2P24 in the wheat rhizosphere

**Fig 1:**
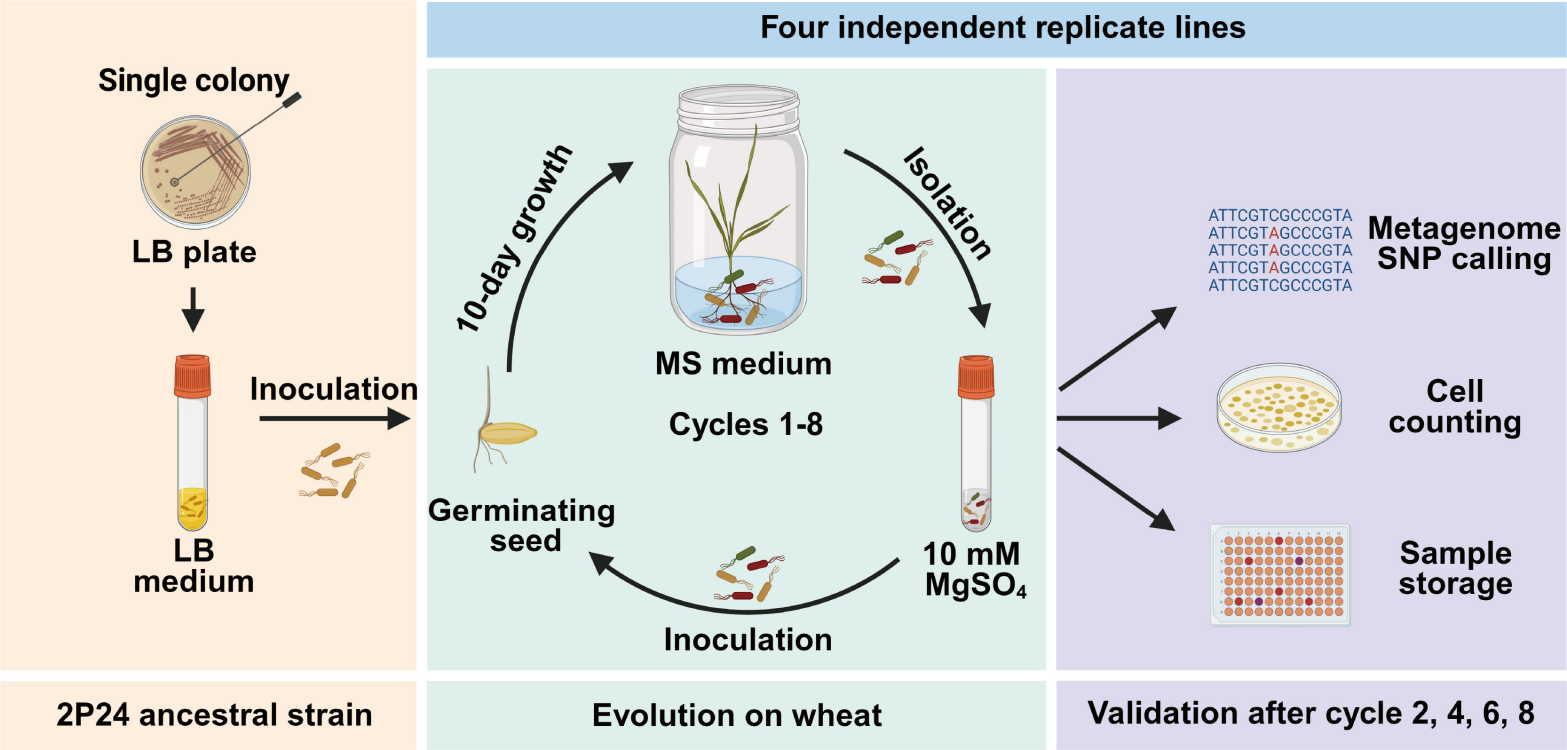
Overview of the experimental design. In this study, we experimentally evolved *Pseudomonas bijieensis* 2P24 in the rhizosphere of sterile wheat plants. We set up a gnotobiotic, carbon-free artificial solid MS medium system to make bacterial fitness strictly dependent on their interaction with plants. We set up four independent replicate lines, which were passed over eight plant growth cycles. Briefly, 10^5^ cells of the original *P. bijieensis* 2P24 population (later: “ancestor”) were introduced to the rhizosphere of a surface-sterilized wheat germinating seed grown in sterile solid MS medium. Each plant growth cycle lasted for 10 days. At the end of each cycle, the rhizosphere bacterial population was isolated and inoculated to a new sterile seed. The remaining bacteria were plated on lysogeny broth (LB) medium for counting cells and stocking 300 evolved colonies at −80°C after SPEs. Rhizosphere bacterial population isolates were sequenced metagenome and called single-nucleotide polymorphism (SNP) in each replicate line at the end of cycles 2, 4, 6, and 8. This image was created using BioRender (http://www.biorender.com).

The experimental evolution of *Pseudomonas bijieensis* 2P24 was conducted through serial passage experiments (SPEs) in the wheat rhizosphere. SPEs achieved the transfer of 2P24 colony-forming units (CFUs) from one plant to another through standardized rhizosphere bacterial harvesting and inoculation protocols. The evolutionary system was set up using a sucrose-free Murashige and Skoog (MS) medium, maintaining a gnotobiotic environment that ensured selection pressures were exclusively mediated by the wheat roots. Initially, approximately 10^5^ CFU of the 2P24 ancestral strain were inoculated onto surface-sterilized, germinating wheat seed and cultivated in a sterile substrate for a period of 10 days (Fig. S2C; Table S1). Subsequently, the bacteria were recovered from the rhizosphere using a 10 mM MgSO_4_ solution and used to inoculate subsequent wheat seed generations. Statistical analysis across SPEs revealed an average inoculum load of ~10⁵ bacterial cells per seed, with a minimum observed inoculation level of 9.33 × 10³ cells per seed. Four biological replicates (lines 1–4) of SPEs were conducted in parallel, yielding four distinct populations, each derived from the original 2P24 ancestral clone (Fig. 1).

In the eight 10-day growth cycles of the SPEs, there was no significant difference in wheat growth status among the four evolved lines. The average estimated number of bacterial generations at each SPE was 9.30  ± 1.13 (mean ± SD) (Table S1), totaling about 80 generations of evolution in the wheat rhizosphere. It is important to note that this calculated generation number is likely an underestimate, as it does not account for the potential mortality of a subset of bacteria in the rhizosphere. Throughout the experimental evolution, the 2P24 strain persistently colonized the wheat rhizosphere without intermediate culturing on artificial media, indicating that the primary selective forces were inherently derived from the wheat roots themselves. Consistent growth and health of the Heshangtou (HST) wheat lines were maintained throughout the experiment, with a sufficient quantity of bacteria (more than 10^6^ CFU) harvested from the rhizosphere at each SPE and used to inoculate the subsequent generation. Standardizing the bacterial inoculation amount across each SPE facilitated the observation and analysis of bacterial population density dynamics within the wheat rhizosphere.

### Genomic variation of *P. bijieensis* 2P24 during 80-day adaptive colonization in the wheat rhizosphere

**Fig 2:**
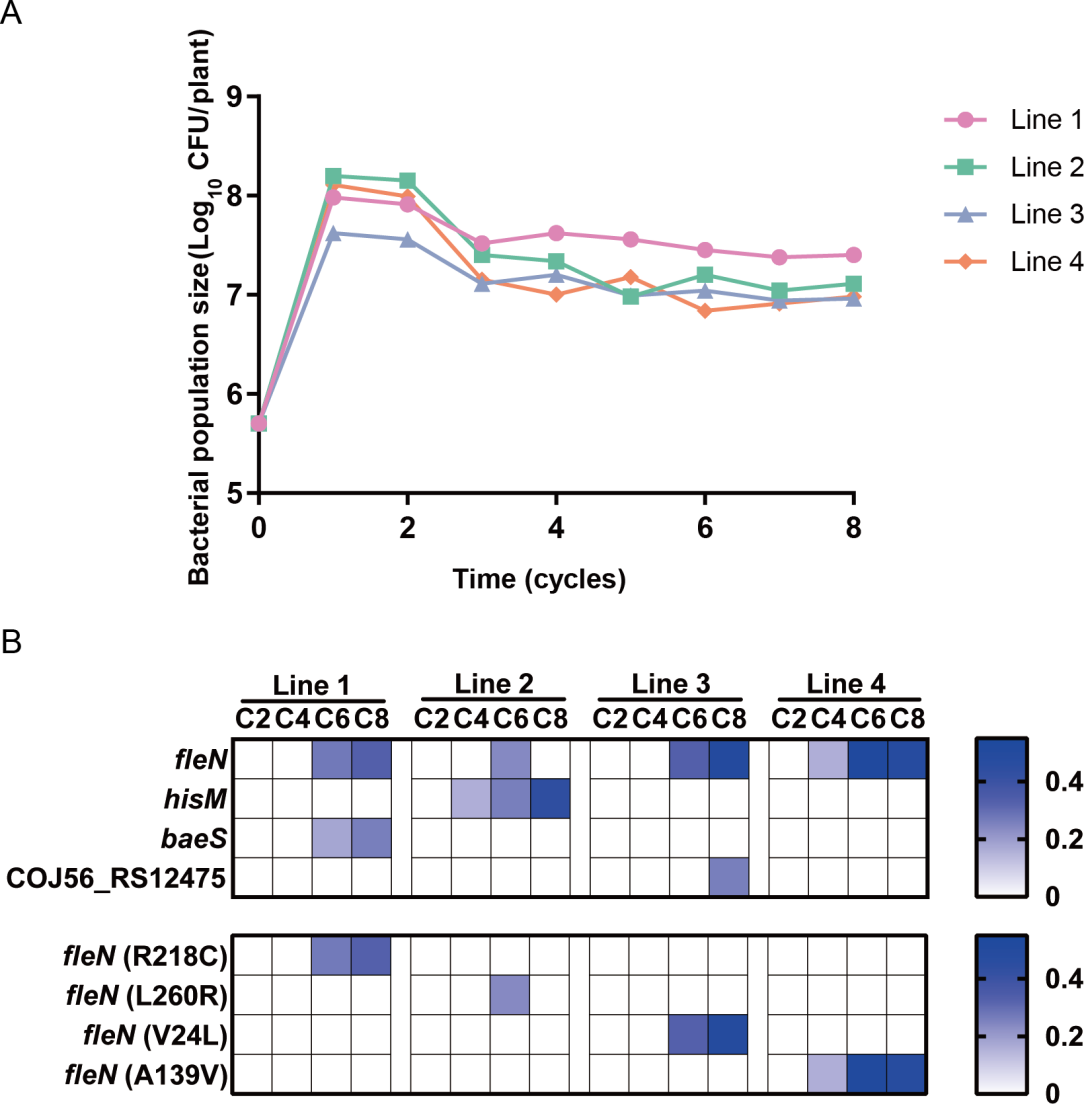
The evolutionary trajectory of *P. bijieensis* 2P24 during adaptive colonization in the wheat rhizosphere. (**A**) Population density dynamics of *P. bijieensis* 2P24 in the wheat rhizosphere were similar in four independent replicate lines. *P. bijieensis* 2P24 was grown in wheat roots in sterile MS medium free of organic carbon for 80 days with 10-day serial transfers. Subsamples were stored at every transfer and used to determine population densities as cell counts per plant. (**B**) The heatmap shows that a total of seven single-nucleotide mutations were found in four lines, with multiple genes experiencing mutations in different evolved lines. Each square represents the frequency of gene mutations in different evolved lines and cycles. From light to deep blue, a frequency range from low to high is shown.

Utilizing a previously described experimental evolution system, we subjected *P. bijieensis* 2P24 to an 80-day adaptive colonization period in the wheat rhizosphere to emulate its natural evolution process. We collected and stored evolved populations from the wheat rhizosphere at 20, 40, 60, and 80 days (cycles 2, 4, 6, and 8, respectively) across four lines of our experimental evolution system (lines 1–4) (Fig. 1). Monitoring the growth dynamics over the passage period by assessing viable bacterial cell counts in the rhizosphere for each cycle revealed that the four populations adapted in similar yet subtly distinct ways. Initially, in the wheat rhizosphere, three out of four 2P24 populations reached peak counts of approximately 10^8^ CFU. After a 20-day adaptation period, population sizes across all lines decreased slightly and stabilized at around 10^7^ CFU, indicating that the 2P24 population has gradually adapted to the rhizosphere environment and established ecological balance in the wheat rhizosphere (Fig. 2A). The establishment of an evolutionarily stable state of bacterial population suggests the potential emergence of key adaptive mutations that restructured the community, thereby stabilizing population size.

To determine the mutations in each line and at each cycle that may lead to adaptive phenotypes, we performed whole-genome resequencing on cells harvested after every two cycles in all populations of the four lines. Given the likelihood of multiple genotypes present in the rhizosphere at any given time, we sequenced the entire population rather than isolating single clones. This approach allowed for a more precise identification of variants within each population. Employing the Illumina MiSeq platform, we performed deep sequencing of the evolved populations to track genomic changes over time. Additionally, we sequenced the 2P24 ancestral strain (ancestor) and performed reference-guided scaffold assembly based on the National Center for Biotechnology Information (NCBI) reference genome. We then mapped the sequence reads of the evolved populations against the ancestral genome to detect variants (22). Our single-nucleotide polymorphism (SNP) calling analysis, complemented by an analysis of allele frequencies, revealed a total of seven SNPs that occurred across populations of the four independent replicate lines after 80 days of selection (Fig. 2B; Table S2). We validated these mutation sites based on PCR and Sanger sequencing. No instances of multiple SNPs co-occurring in a single strain were observed (Fig. S1). As early as 20 days into the evolution, adaptive mutations began to emerge in the evolved populations, with the *fleN* gene exhibiting mutations across different population lines and accumulating over time, suggesting potential parallel evolution. Unique mutations that only occur within a single evolved line, such as *baeS*, *hisM*, and COJ56-RS12475 gene mutations, are speculated to be the result of random events during the evolutionary process or may be the result of selection under specific conditions, such as slight differences in experimental conditions across different evolved lines (Fig. 2B). The flagellar number-related gene *fleN* exhibited mutations in all four evolved lines, with mutation frequencies reaching as high as 49% in the later stages of evolution. This observation leads us to hypothesize that mutations in the *fleN* gene are closely associated with the colonization of 2P24 in the wheat rhizosphere.

### Accumulation of *fleN* mutants in evolved populations enhances the colonization capacity of 2P24 in the wheat rhizosphere

**Fig 3:**
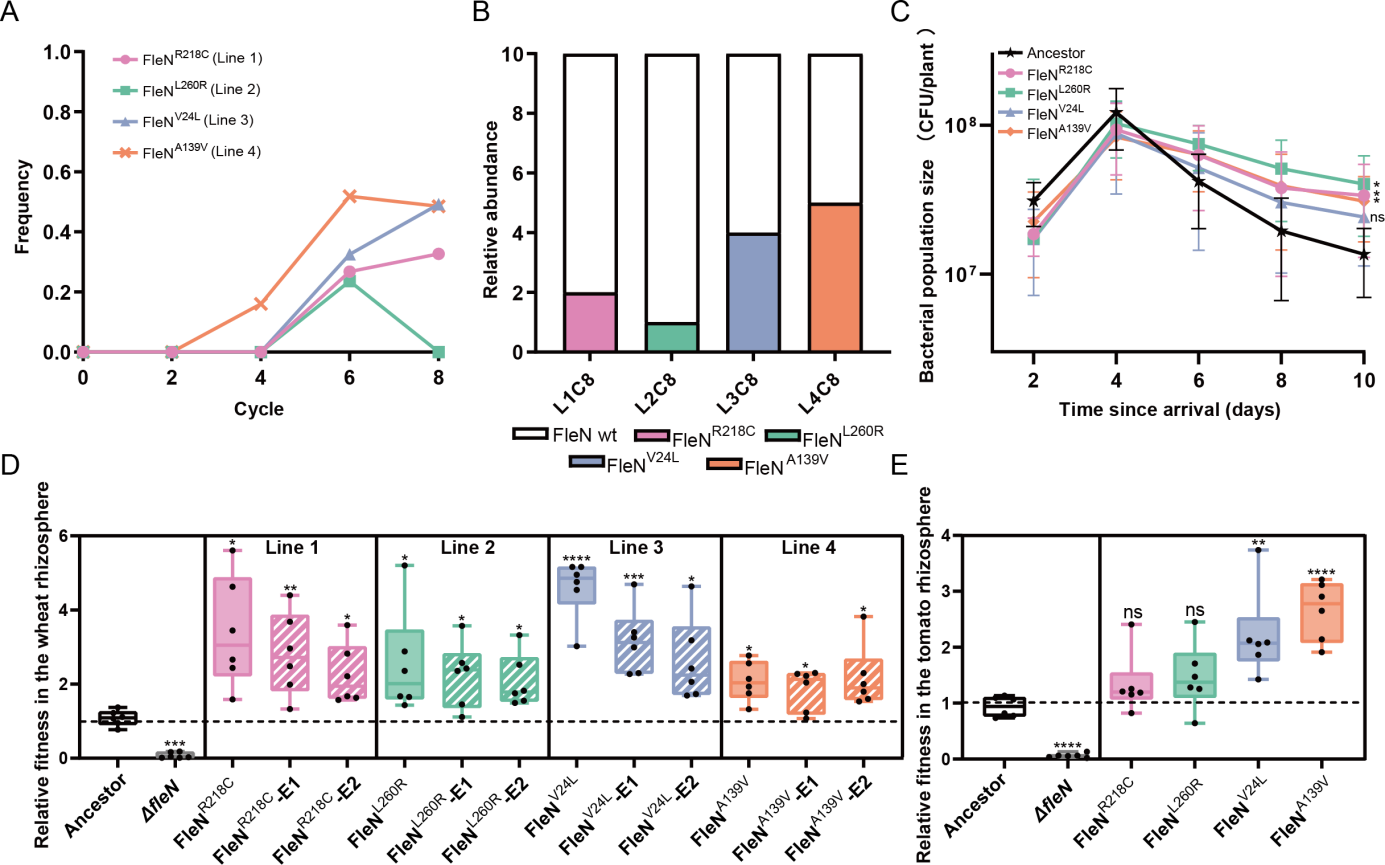
The *fleN* mutants have high relative abundance at the end of the evolutionary experiment in four lines and have high fitness in the wheat rhizosphere. (**A**) The frequency of *fleN* mutations in four independent evolved lines at cycles 2, 4, 6, and 8 was calculated by dividing the number of reads with a non-reference allele by the total number of reads for that nucleotide. (**B**) The relative abundance of *fleN* mutants at the end of evolution in four lines. Ten stored strains in the eighth cycle (C8) of every line were randomly selected to verify the number of each *fleN* mutant. (**C**) Life history in the absence of competition in the wheat rhizosphere. Population sizes of *P. bijieensis* 2P24 ancestral strains and four *fleN* mutant strains after inoculation via flooding. (**D**) Box plot of the relative fitness of the control ancestral 2P24 clone with the constructed and evolved *fleN* mutant clones on wheat roots. FleN^R218C^, FleN^L260R^, FleN^V24L^, and FleN^A139V^ are constructed *fleN* mutant strains. FleN^R218C^-E1, FleN^R218C^-E2, FleN^L260R^-E1, FleN^L260R^-E2, FleN^V24L^-E1, FleN^V24L^-E2, FleN^A139V^-E1, and FleN^A139V^-E2 are evolved *fleN* mutant strains isolated and stored from four final evolved populations after SPEs. (**E**) Box plot of the relative fitness of the control ancestral 2P24 clone with the constructed *fleN* mutant clones in the tomato rhizosphere. All the strains were competed by 2P24-Km (2P24 carrying a Km resistance cassette). The relative fitness was calculated based on the deviation from the initial 1:1 genotype ratio (dashed line) after direct competition. A minimum of six replicates were performed for each clone. Fitness values above the dashed line indicate a higher competitive advantage of *fleN* mutants relative to their ancestral genotypes without *fleN* mutations, whereas values below the dashed line denote the decreased competitive ability of evolved *fleN* mutants. Solid box: constructed *fleN* mutant strains; dotted box: evolved *fleN* mutant strains. Different colors represent different evolved lines. Statistical analyses were performed using two-tailed Student’s *t*-test in panels **D** and **E**. *P < 0.05, **P < 0.01, ***P < 0.001, ****P < 0.0001.

After 80 days of adaptive evolution in the wheat rhizosphere, the flagellar number-related gene *fleN* (20, 21) underwent mutations in all evolved lines, with the proportion of *fleN* mutants accumulating over the course of evolution in three out of four lines. This observation suggests that mutations in the *fleN* gene play a significant role in the adaptation of 2P24 to the wheat rhizosphere. The specific mutation sites within the *fleN* gene varied across the four independent replicate lines (Table S2). By conducting SNP calling analysis on whole-genome sequencing data, we quantified the mutation frequency of the *fleN* gene within each sequenced evolved population, calculating the frequency of allelic gene mutations (Fig. 3A). To verify the accuracy of this method, we randomly selected 10 single colonies from each of the four independently evolved 2P24 populations after 80 days and performed PCR detection on the mutation sites of the *fleN* gene (Fig. 3B). The results revealed that the actual mutation frequency within the community (R218C: 20%, L260R: 10%, V24L: 40%, and A139V: 50%) was largely consistent with the results obtained from the SNP analysis (R218C: 33%, L260R: 0%, V24L: 49%, and A139V: 48%). The abundance of the L260R mutant in the final evolved population is very low, which may be caused by the random evolutionary pressure that exists only in line 2. This pressure selected and enriched more *hisM* mutants, which may be more conducive to colonization of the entire 2P24 population in line 2 (Fig. 2B). We subsequently constructed mutants FleN^R218C^, FleN^L260R^, FleN^V24L^, and FleN^A139V^ using homologous recombination to verify the contribution of four point mutations occurring on the *fleN* gene to colonization ability. The growth and development of wheat is a dynamic process, during which most *fleN* mutants demonstrated significant adaptive advantages at the late stage (day 10 post-inoculation) of mono-colonization in the wheat rhizosphere (Fig. 3C). Furthermore, competitive colonization experiments between *fleN* mutants and the ancestor revealed that both artificially constructed *fleN* mutants and those isolated from evolved populations exhibited improved colonization capacity relative to the ancestor, with an enhancement ranging from 2.1 to 4.6 times (Fig. 3D). In addition, both the *fleN* mutant strains and the ancestral strain have no effect on wheat growth (Fig. S2). The evolved FleN^R218C^ and FleN^V24L^ (E1 and E2) mutant strains exhibited slightly reduced adaptive colonization capacity in the wheat rhizosphere compared to their artificially constructed FleN-SNP mutant strains (Fig. 3D). This phenomenon may stem from evolutionary trade-offs in the evolved strains, where long-term adaptation to the rhizosphere environment potentially involved balancing colonization capacity with other unidentified adaptive phenotypes. These cryptic phenotypic adaptations warrant further investigation. Notably, the mutants FleN^V24L^ and FleN^A139V^ exhibited more general adaptability. These strains also had significantly enhanced competitive fitness in the tomato rhizosphere compared to the ancestor (Fig. 3E; Fig. S4).

The *baeS* and *hisM* mutant strains isolated from the final evolved populations of lines 1 and 2, respectively, showed no significant difference in competitive colonization compared to the ancestral strain in the wheat rhizosphere (Fig. S5B). These two mutations are likely neutral, as they did not significantly affect the fitness of mutant strains and may be random events during evolution. Furthermore, because they cannot provide significant adaptive advantages, these mutations were not selected in other evolved lines. In addition, the motility, biofilm production ability, and growth ability in wheat root exudates of the evolved *baeS* and *hisM* mutant strains were consistent with the ancestor (Fig. S6 and S8). In contrast, the COJ56_RS12475 mutant strains from line 3 demonstrated a slightly reduced competitive colonization capacity than the ancestor (Fig. S5B). Since the COJ56_RS12475 gene lacks functional annotation and its evolved mutants show no significant differences in colonization-related phenotypes compared to the ancestral strain, we speculate that this mutation may make the entire evolved population more adaptable but not dominant in competitive colonization (1:1) with the ancestral strain. Collectively, these findings confirm that the accumulation of *fleN* mutations in evolved populations significantly bolsters the colonization of 2P24 in the plant rhizosphere. Additionally, the colonization fitness benefits conferred by *fleN* mutations appear to be specific to the rhizosphere (Fig. S5A).

### The *fleN* mutations play a crucial role in modulating flagellar phenotypes and associated traits

**Fig 4:**
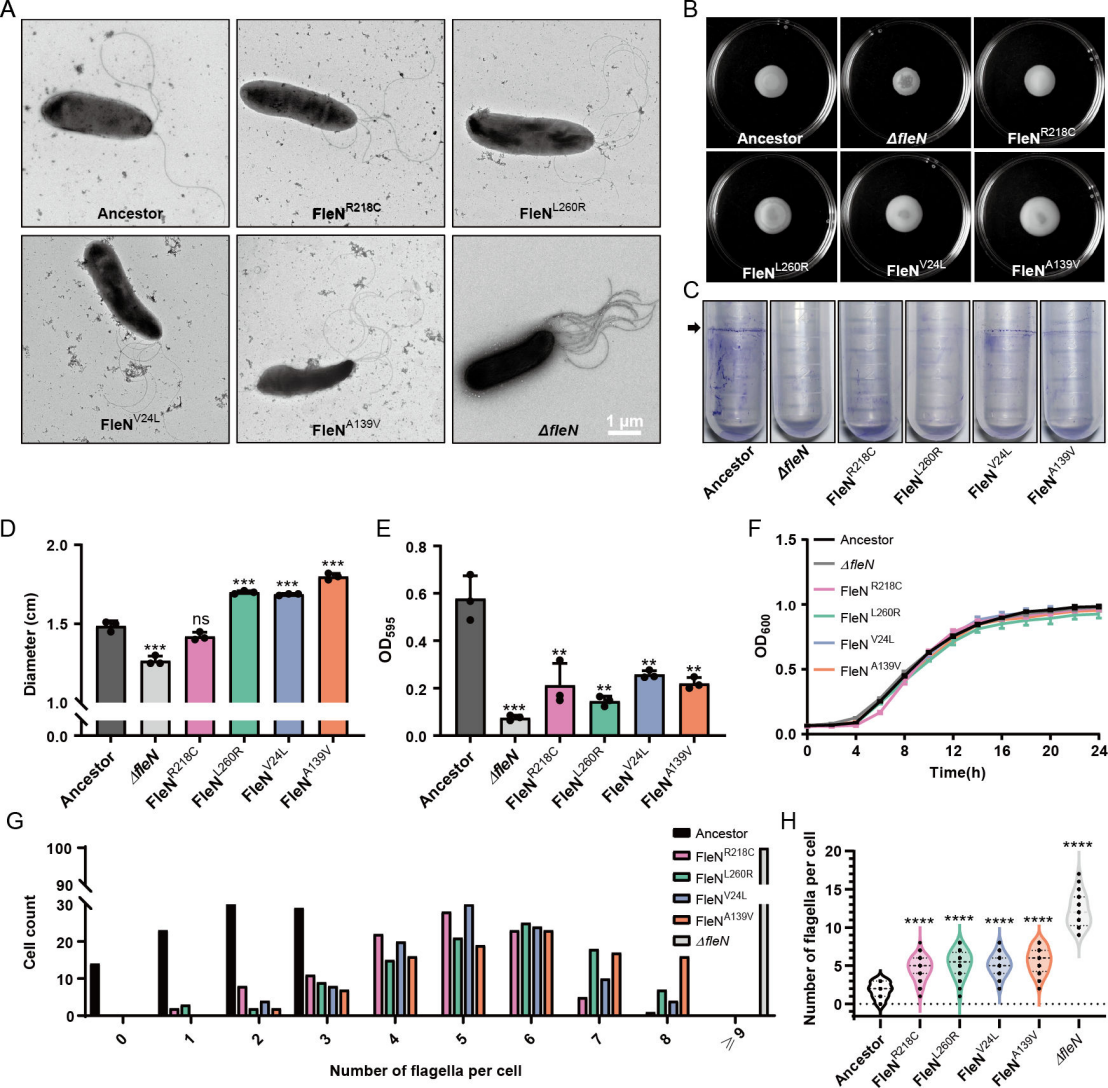
The *fleN* mutant strains moderately increased the number of flagella and showed altered biofilm formation and motility. (**A**) Electron micrographs showing flagellar phenotypes of different strains, including the *P. bijieensis* 2P24 (ancestor), the knockout strain (Δ*flen*), and the constructed SNP mutants (FleN^R218C^, FleN^L260R^, FleN^V24L^, and FleN^A139V^). (**B**) The swarming motility of the *P. bijieensis* 2P24 ancestral strain (ancestor), the knockout strain (Δ*fleN*), and the constructed *fleN* mutant strains (FleN^R218C^, FleN^L260R^, FleN^V24L^, and FleN^A139V^) is shown on LB medium with 0.45% soft agar. The swarming zone was calculated by measuring the diameters of the colonies. (**C**) The biofilm formation of the *P. bijieensis* 2P24 ancestral strain (ancestor), the knockout strain (Δ*fleN*), and the constructed *fleN* mutant strains (FleN^R218C^, FleN^L260R^, FleN^V24L^, and FleN^A139V^) is shown. (**D**) Bar chart represents the swarming zone for different strains of *P. bijieensis* 2P24. The *y*-axis shows the diameter of the swarming zone in centimeters. (**E**) Spectrophotometric quantification of biofilms formed by *P. bijieensis* 2P24 ancestral strain and its constructed *fleN* mutants. Mean values of three replicates are given, and error bars indicate standard errors in panels **D** and **E**. Different colors represent different evolved lines in panels **D** and **E**. Visual representation of biofilm formation by the *P. bijieensis* 2P24 ancestral strain and its constructed *fleN* mutants on tubes is shown. Biofilms were stained with crystal violet and determined at 595 nm after 20 h of growth using a spectrophotometer. (**F**) Growth of the *P. bijieensis* 2P24 ancestral strain and its constructed *fleN* mutants in LB medium. Bacterial yield was determined as the maximum optical density at 600 nm per 2 h of growth using a spectrophotometer. Mean values of three replicates are given, and error bars indicate standard errors. (**G**) Quantification of the number of flagella per cell in the *P. bijieensis* 2P24 ancestral strain and its constructed *fleN* mutants (see panel** A** for corresponding electron micrographs). (**H**) Statistical graph of the flagellar number of the ancestor and its constructed *fleN* mutants. Count 100 cells per strain in panels** G** and **H**. Statistical significance of all the bar charts (*P* value) was calculated using unpaired Student’s *t-*test. SD is calculated from three independent experiments in panels** D** and **E**. **P < 0.01, ***P < 0.001, ****P < 0.0001.

The *fleN* gene is recognized as a key determinant of the bacterial flagellar number (20, 21). We examined the flagella of the 2P24 ancestral strain and its *fleN* mutants under transmission electron microscopy (TEM) (Fig. 4A). The ancestral strain displayed a maximum of three polar flagella, whereas the *fleN* knockout strain (Δ*fleN*) presented a hyperflagellated phenotype, exhibiting at least nine flagella (Fig. 4A). The Δ*fleN* mutant of 2P24 exhibited the phenotype of hyperflagellated morphology and reduced motility (Fig. 4B and D), which is consistent with the Δ*fleN* mutant phenotype of other *Pseudomonas* spp., such as *Pseudomonas aeruginosa* (21, 23). The evolved strains FleN^R218C^, FleN^L260R^, FleN^V24L^, and FleN^A139V^ of 2P24, adapted to the wheat rhizosphere, showed an intermediate flagellar number range (four to eight) between the ancestor and the Δ*fleN* strains. Notably, FleN^R218C^ and FleN^V24L^ strains predominantly presented five flagella, while the other two strains mostly had six flagella (Fig. 4G and H). Compared to the ancestor, the swarming ability of constructed FleN^L260R^, FleN^V24L^, and FleN^A139V^ strains and its evolved mutant strains significantly improved on lysogeny broth (LB) medium (Fig. 4B and D; Fig. S7A and B). Strains with the FleN^R218C^ mutation showed no significant difference in swarming ability compared to the ancestor on LB medium, but they had stronger colonization ability across the root distances (Fig. S3). We speculate that the adaptive advantage of the FleN^R218C^ mutation is environment dependent, manifested in the complex ecological environment around wheat roots but “masked” in homogeneous, nutrient-rich LB medium. The motility of all constructed point mutant strains was significantly enhanced on the casamino acid medium, with the colony edges exhibiting a lace-like shape (Fig. S7C and D). However, we observed a decrease in the ability of all mutant strains to form biofilms (Fig. 4C and E; Fig. S7E and F), consistent with the phenotype caused by point mutations (V178G) in the *fleN* gene in *P. aeruginosa*. The improvement of motility ability may enable the *fleN* mutants to occupy more distant and expansive ecological niches in the rhizosphere than their ancestors, thereby exhibiting superior colonization ability across the root distances (Fig. S3) (24, 25). Given the similar growth rates between the *fleN* mutants and the ancestor, we attribute the enhanced adaptation of the *fleN* mutants to the wheat rhizosphere primarily to the increased number of cell flagella rather than enhanced growth capacity (Fig. 4F; Fig. S8). Based on these observations, we speculate that during the adaptation process to the wheat rhizosphere, 2P24 underwent point mutations in the flagellar gene *fleN*, resulting in a moderate increase in bacterial flagellar number and significantly enhanced motility. This adaptive mutation is advantageous for bacteria to migrate more rapidly from the oligotrophic environment, where the nutrient source is solely the wheat root, to more nutritious areas, forming bacterial communities with stronger colonization ability.

### The FleN mutants reduce their inhibition of ATPase activity in FleQ, resulting in alterations to the flagellar number in 2P24

**Fig 5:**
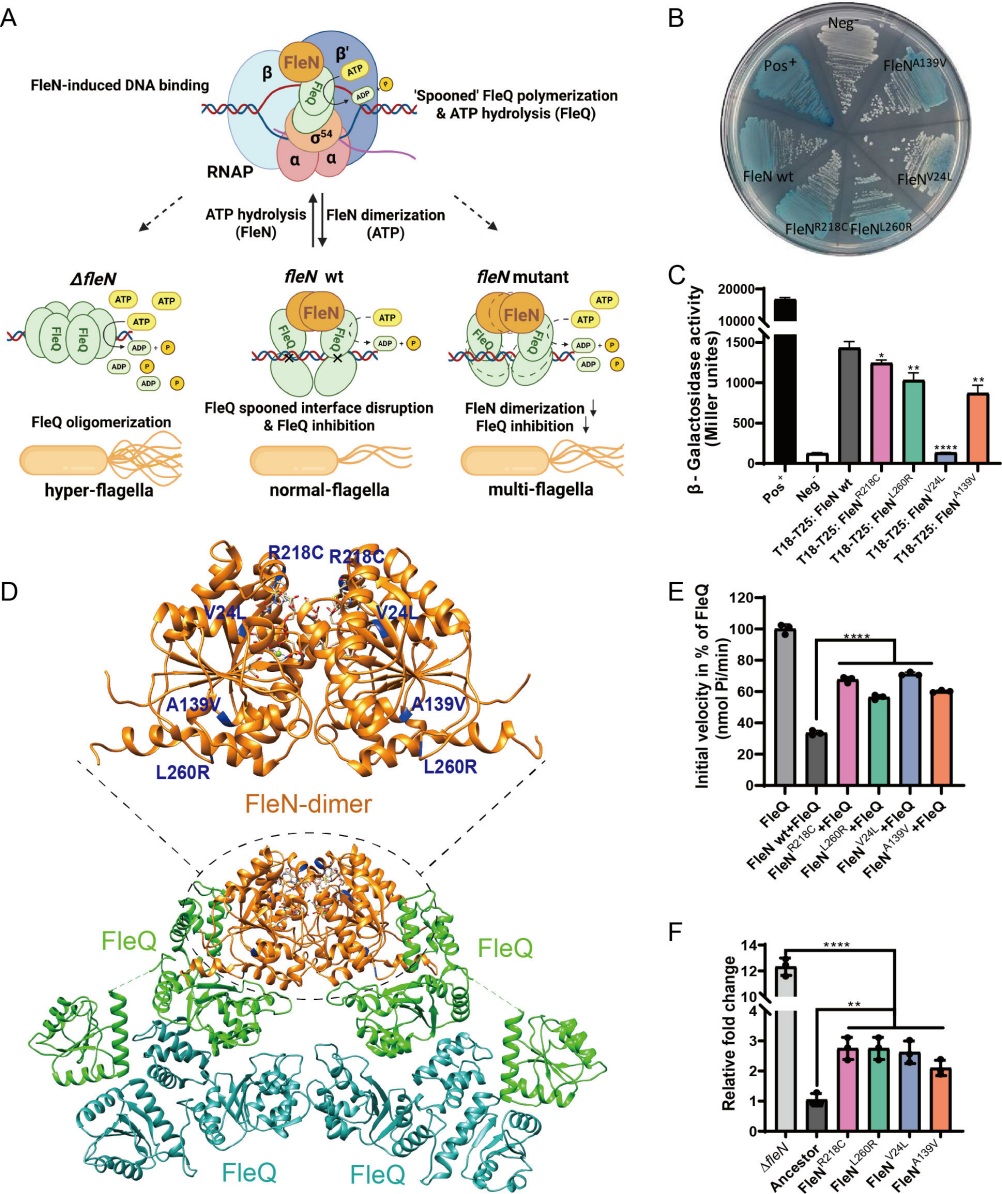
The FleN mutants reduce their inhibition of ATPase activity in FleQ, resulting in changes to the number of flagella in strain 2P24. (**A**) Model for σ^54^-mediated transcription regulation by the antiactivator FleN. The master regulator FleQ is in green. Antiactivator FleN is shown in orange. The correct flagellar number is achieved between fine-tuning ATP-dependent FleQ hydrolysis and σ^54^-dependent transcription activation vs ATP-dependent FleN dimerization and FleQ inhibition. FleN stimulates DNA binding and promotes FleQ oligomerization-dependent transitions of RNA polymerase (RNAP)-promoter DNA complexes from closed to intermediate to open states. In contrast, FleN dimerization inhibits the intermediate-to-open transition and locks FleQ dimers in a catalytically incompetent state. (**B** and **C**) The dimer interactions of FleN wt and its variants *in vivo* were measured by bacterial two-hybrid to detect *β*-galactosidase activity. From light to deep blue, interaction strength from low to high is shown. The plasmids pKT25-zip and pUT18C-zip serve as positive controls for complementation (pos^+^). Empty vectors pKT25 and pUT18C are co-transformed into BTH101 as negative control (neg^−^). (**D**) Predicted FleN-FleQ structure based on the reported complex structures (Protein Data Bank: 8P53 and 7EJW) showing the FleN dimer in orange and its mutant sites in deep blue, interacting with FleQ domains colored green and light blue, forming a 4:2 FleQ:FleN complex. (**E**) FleN inhibits the ATPase activity of FleQ. The ATPase activity of FleQ (1 µM) was measured at 2 mM ATP in the absence and presence of FleN wt and its variants (5 µM). The initial velocities (nmol Pi/min) are plotted in percentage of FleQ. (**F**) Relative transcript levels of *fleS* in various strains of *P. bijieensis* 2P24 compared to the ancestor. Statistical significance of all the bar charts (*P* value) was calculated using unpaired Student’s *t*-test. SD is calculated from three independent experiments in panels **C**, **E**, and **F**. *P < 0.05, **P < 0.01, ****P < 0.0001.

FleQ is a bacterial enhancer-binding protein capable of forming active hexamers that extensively hydrolyze adenosine 5′- triphosphate (ATP) (26, 27). In *Pseudomonas* spp., it regulates flagellar motility and biofilm formation through distinct transcriptional activation mechanisms, achieved by forming complexes with FleN. FleQ binds to the flagellar gene promoter DNA in the form of a “spooned” oligomer, mediated by the weak ATPase FleN, and hydrolyzes ATP while dependent on σ^54^ polymerase for transcriptional activation of a series of flagellar genes. The ATP-dependent FleN dimerization inhibits FleQ’s ATPase activity by disrupting the spooned dimer interface of FleQ and preventing its higher-order oligomerization. Bacteria fine-tune the balance between these two forms of the FleN-FleQ complex to form the correct number of flagella (Fig. 5A) (26–28). We first checked the ATPase activities of FleN mutants (FleN^R218C^, FleN^L260R^, FleN^V24L^, and FleN^A139V^). The mutants showed significantly different ATPase activities compared to the wild-type protein, indicating that these four mutation sites play a crucial role in maintaining the normal ATPase activity of FleN protein (Fig. S9C). Bacterial two-hybrid experiments analyzing the dimerization capabilities of wild-type FleN (FleN wt) and its mutants revealed a significant reduction in dimer formation for four FleN mutants compared to the wild-type protein (Fig. 5B and C). Moreover, compared to FleN wt, which completely abolished FleQ ATPase activity, the FleN mutants all exhibited reduced ability to inhibit the activity of FleQ with 56.4%–71.2% residual activity (Fig. 5E). These results suggest that the reduced dimerization capability of FleN point mutants leads to the decreased inhibition of ATPase activity on FleQ, thereby causing bacterial enhancer-binding protein FleQ to overactivate the transcription of some flagellar genes, resulting in a moderate increase in the number of flagella in the 2P24 FleN mutants adapted to the wheat rhizosphere.

### Interaction between FleQ and FleN mutants is essential for transcription regulation

Previously, it was known that the expression of genes of the two-component system *fleSR*, which are part of the flagellar cascade, is directly activated by FleQ (26). We performed a real-time quantitative polymerase chain reaction (RT-qPCR) experiment to assess the effect of FleN mutations on the transcription regulation by FleQ. The *fleN* knockout (Δ*fleN*) strain showed higher levels of expression of the *fleS* and *fleR* genes as compared to the 2P24 ancestral strain (Fig. 5F; Fig. S9D). The expression levels of the *fleS* and *fleR* genes in the four point mutation strains of *fleN* were intermediate between the ancestor and the Δ*fleN* strain. These results indicate that the FleN mutants regulate the transcription of bacterial flagellar genes appropriately by altering their interaction with FleQ.

In summary, combining previous research, we believe that the moderate increase in the 2P24 flagellar number is due to the reduced strength of ATP-dependent dimerization of FleN point mutations (Fig. 5B and C), leading to a decreased inhibition of FleQ’s ATP hydrolysis activity (Fig. 5E), resulting in increased transcription levels of key flagellar genes such as *fleS* and *fleR*.

## DISCUSSION

In this study, we employed an evolutionary approach combining experimental evolution with phenotypic and comparative genomics to analyze the adaptive potential of *Pseudomonas bijieensis* 2P24 in the wheat rhizosphere. It is recognized that key genes necessary for colonization are not typically subjected to deletion mutations within the rhizosphere; rather, they may undergo nucleotide sequence alterations to better adapt to the rhizosphere. Such as in our evolutionary experiment, compared to the hyperflagellated phenotype of *fleN* deletion mutants, the flagella of FleN-SNP strains in this study were subtly adjusted to four to eight flagella, generally enhancing motility. This is in stark contrast to the complete loss of motility observed in *fleN* deletion mutants (Fig. 4A) (21, 23). Such adaptive changes can vary under different conditions, such as different plants or soils. Traditional molecular genetic techniques, including transposon mutagenesis and *in vivo* expression technology, may struggle to capture these subtle adaptive changes (6, 29, 30). In contrast, experimental evolution can simulate the natural evolutionary process of bacteria in the rhizosphere, revealing more nuanced adaptive molecular mechanisms, such as SNPs and small insertions or deletions (indels). This approach avoids the limitations of genetically deficient phenotypes that do not reflect adaptability and circumvents the influence of redundant genetic factors. With the advent of high-throughput sequencing technologies, such as genome sequencing, amplicon sequencing, and transposon sequencing, comparative omics analyses of species, functional genes, and expression levels under rhizospheric and non-rhizospheric conditions can unearth important colonization-related factors (7, 9, 31). However, these methods often necessitate the collection of extensive genomic information from closely related microbes for bioinformatics analysis (32). Moreover, rhizosphere colonization is a dynamic process in which bacteria continuously adapt to the rhizosphere. Compared to common research methods that focus on data collection and study at a fixed spatiotemporal point, experimental evolution allows for continuous observation and tracking of bacterial genomic and phenotypic changes, thereby uncovering underlying mechanisms. During pivotal periods of the evolutionary experiment, we sequenced not only a few evolved bacterial isolates but also the entire bacterial community from the rhizosphere. This comprehensive approach enabled us to identify mutations that are frequent, stable, and crucial for adaptation and colonization within the entire community.

Although some experimental evolution studies have focused on adaptive changes in microbial populations in the plant rhizosphere, there are relatively few studies conducting such adaptive evolution experiments with PGPRs inoculated in the plant rhizosphere (15, 33). Here, we investigated the changes in *Pseudomonas bijieensis* 2P24 after long-term selection over eight plant growth cycles (80 days) on its host plant, wheat, with a focus on understanding the adaptive colonization molecular mechanism of 2P24 in the rhizosphere (Fig. 1). After 40 days of adaptation to the rhizosphere, SNPs occurred and accumulated in genes related to motility, amino acid transport (34), and environmental sensing (35) (Fig. 2). The parallel mutations of genes occurring in different evolved populations and accumulating over time lead us to believe that these genetic alterations play a crucial role in the adaptation and colonization of bacteria in the wheat rhizosphere (36).

Our experimental evolution study expands the current understanding of the adaptive colonization of biocontrol *Pseudomonas* in the rhizosphere, as previously explored by Li et al., who reported significant improvements in the adaptability of *Pseudomonas protegens* CHA0 after 6 months of evolution in the *Arabidopsis* rhizosphere. Parallel single-nucleotide mutations in the global regulatory factors *gacS* and *gacA* played a significant role in modulating bacterial competition and interactions with plants. Additionally, mutations in genes related to motility (*flhA* and *fleQ*) and cell surface structures (OBC3 gene cluster) also occurred (33, 37, 38). These findings differ from the evolved loci identified in our study, indicating that different bacterial strains, crops, soil matrix characteristics, and microbial community structures may impose varying selective pressures during adaptation to the rhizosphere, potentially leading to different genetic mutations. Thus, experimental evolution studies tailored to specific conditions hold great promise for obtaining improved bacterial strains with significantly enhanced colonization ability and biocontrol efficacy under those particular conditions.

The correct number of flagella is essential for motility and is associated with biofilm formation and the biocontrol effect of biocontrol bacteria (39). The mechanism for maintaining flagellar number is not yet fully understood, but the interaction between flagellar proteins FleN and FleQ seems to have evolved to meet specific requirements of the various patterns (20, 26, 39). This variability underscores the importance of the correct flagellar pattern for motile species. In this study, minor mutations on the highly conserved negative flagellar number regulator FleN allowed for the enhancement of competitive advantages in the wheat rhizosphere (39). Bacteria initially inoculated onto wheat seeds ultimately colonized the entire root system through adaptive evolution, during which enhanced motility was selected. The *fleN* point mutation conferred superior motility to bacterial cells, enabling them to occupy more distant and expansive ecological niches in the wheat rhizosphere, particularly in nutrient-rich distal root regions. Furthermore, the SPEs strengthened this selection. Strains with stronger mobility could more rapidly occupy optimal root niches during each reinoculation cycle, thereby driving the gradual accumulation of *fleN* mutants in the evolving population (Fig. 2B; Fig. S2C and S3) (2). The flagellar number regulation system of 2P24 is consistent with that reported in *P. aeruginosa* and other γ-proteobacteria (Fig. 5) (21, 23). We speculate that the *fleN* gene, a widely present and conserved regulator of flagellar numbers in *Pseudomonas*, facilitates bacterial adaptation to various environments. When environmental conditions change, bacteria rapidly mutate in this gene to balance motility and biofilm production abilities, allowing them to adapt to new environments and colonize extensively (Fig. S10) (24, 25). This study shows that experimental evolution is an excellent selection method that follows bacterial adaptation to the environment, spontaneously producing beneficial mutations with high environmental specificity and accuracy, restoring the real evolutionary process of bacteria in natural environments. This experimental evolution system is profoundly significant for selecting and modifying biocontrol bacteria, as well as for studying microbial environmental adaptability.

## MATERIALS AND METHODS

### Bacterial strain and growth conditions

For our experimental evolution, we used *Pseudomonas bijieensis* 2P24 as a model strain, which was initially isolated from a wheat take-all decline soil in China. The strain was chromosomally tagged with GFPuv and a tetracycline resistance cassette to enable specific tracking of the strain and detection of contaminations (40). Prior to the experiment, bacteria were grown for 48 h on an LB agar plate supplemented with 50 µg/mL tetracycline. A single colony was randomly picked and grown for 16 h in LB at 28°C with agitation. The cell culture was then washed three times in 10 mM MgSO_4_ and adjusted to 10^7^ cells/mL and used as inoculant for all plants. This inoculant was also stored at −80°C as frozen ancestral stock, from which “ancestor” isolates were picked in later experiments.

### Host plant and growth conditions

We used wheat genotype HST as a model host plant. After disinfecting the air-dried wheat seeds with a 5% sodium hypochlorite solution for 30 min, the residual sodium hypochlorite was removed by washing with sterile water, and the surface-sterilized seeds were put on sterile filter paper in Petri dishes with 1.5 mL ddH_2_O. The petri dishes were placed at room temperature in the dark for seed germination. After 36 h, the germinating seeds were sown in closed and sterile tissue culture bottles (diameter: 10 cm, height: 20 cm) with agar-solidified (0.7% agar [wt/vol]) MS basal salts medium (Caisson Labs) for the selection experiment. One hundred milliliters MS basal salts medium was added to each bottle.

### Design of the evolutionary experiment

We evolved *P. bijieensis* 2P24 in the wheat rhizosphere to establish an adaptive evolution system in the laboratory. We set up four independent replicate lines, which were grown for eight independent plant growth cycles (Fig. 1). To start the experimental evolutionary experiment, we first grew *P. bijieensis* 2P24 on an LB agar plate. Then we inoculated a single colony of *P. bijieensis* 2P24 into 3 mL

of LB liquid medium to grow for 16 h at 28℃. Some of these cells were stored as the ancestral 2P24 (abbreviated as ancestor) and compared all further phenotypic and genomic properties of the evolved populations with this ancestor. Other cells were washed, and the cell concentration was diluted to 10^7^ CFU/mL with 10 mM MgSO_4_ for soaking the germinating wheat seeds (each seed is approximately 0.8 × 0.5 × 0.4 cm^3^ in size) (Fig. S2C). After soaking for 30 min, the average bacterial load per wheat seed was approximately 10^5^ bacterial cells. We sowed the germinating seeds in carbon-free MS agar medium within the sterile tissue culture bottles (just one plant per independent replicate line). Inoculated plants were then grown for 10 days (22°C, 10 h light/14 h dark, light intensity 100 µmol/m^2^/s), after which the plant growth cycle was terminated, and the root-associated bacteria were harvested by placing the roots of plants into 5 mL Eppendorf tubes filled with 2 mL 10 mM MgSO_4_. Rhizosphere bacteria were suspended into the liquid using a VOTEX-GENIE 2 (Scientific Industries) at SHAKE10 for 30  min. Newly germinated wheat seeds were then soaked in 2 mL of bacterial suspension for 30 min before being planted in carbon-free MS agar medium to initiate the next plant growth cycle. Harvested rhizosphere bacterial suspensions were serially diluted and plated for CFU enumeration. Statistical analysis across eight cycles revealed an average inoculum load of ~10⁵ bacterial cells per seed, with a minimum observed inoculation level of 9.33 × 10³ cells per seed. Possible contaminations were checked by plating the suspension on LB agar plates, and it was verified that all colonies carried the GFP marker gene, as observed under UV light. The remaining bacteria were plated on LB medium for counting cells and stocking 300 evolved colonies at −80°C after SPEs. Rhizosphere bacterial population isolates were sequenced metagenome and called SNP in each replicate line at the end of cycles 2, 4, 6, and 8.

### Construction of the *fleN* gene mutant

The *fleN* mutants were constructed using a two-step homologous recombination strategy (41). The primers used for construction are listed in Table S3.

### Transmission electron microscopy

Samples for electron microscopy were prepared according to standard methods. Cells were grown to mid-exponential phase on the LB agar plate. We suspended a single colony in 20 µL of ddH_2_O, and the cell suspension was applied to glow-discharged and carbon-coated copper grids (400 square mesh, Plano). The samples were washed immediately once with ddH_2_O and were negatively stained with uranyl acetate (2%) for 10 s. Electron microscopy was performed on a TEM HT7700 (Hitachi Ltd.) at 120 kV. One hundred cells were randomly selected from each sample under the microscope, and their flagellar numbers were counted.

### Bacterial life-history trait measurements

#### Motility

Bacterial motility was assessed by inoculating 5 and 3 µL of the same concentration (OD_600_ = 1) of bacterial solution on LB and casamino acid medium with 0.45% agar plate (42, 43).

#### Biofilm formation

Biofilm formation was quantified using an improved standard protocol (44).

#### Growth curve

All the bacterial isolates were grown in 100-well plates with 200 µL LB liquid medium at 28°C with 180 rpm shaking. Bacterial yield was determined as the maximum optical density at 600 nm after 2 h of growth using an automated microbiology growth analysis system (Bioscreen C).

#### Whole-genome deep sequencing and variant calling analysis

Genomic DNAs of rhizosphere *P. bijieensis* 2P24 population isolates were extracted using the standard method (45). Whole-genome sequencing was performed by Beijing Saimo Baihe Biotechnology Co., Ltd. (Beijing, China), using the Illumina HiSeq 2500 sequencing platform to obtain an approximate calculated level of coverage of more than 300× for each evolved population. We first aligned the reads from the ancestral strain to the deposited *P. bijieensis* 2P24 genome (NCBI RefSeq assembly: GCF_002865505.1) in the NCBI nucleotide sequence database, thereby constructing and updating the reference genome of 2P24 that carried the GFPuv marker gene on its chromosome. We mapped reads of the evolved populations against the ancestral 2P24 genome using BWA (46). We referred to the variant calling method to identify and functionally annotate SNPs and small indels for each individual strain (47). The assembled genome sequencing data for this study are deposited at the NCBI database (under Sequence Read Archive: SRS20259179 and PRJNA1069306).

#### Verification of point mutation frequency by PCR

The mutation frequency of the *fleN* gene in the 2P24 evolved population was verified using the PCR method. Amplification of the *fleN* gene was performed by Thermocycler (Eppendorf, Germany) using primers *fleN*-F and *fleN*-R (Table S3).

#### Relative competitive fitness of *fleN* mutants measured *in vivo* and *in vitro*

The relative competitive fitness of selected *fleN* mutants was measured in direct competition with their direct ancestors both in the plant rhizosphere and in LB medium. The relative fitness was performed using a mixed inoculum consisting of equivalent CFU (1:1 ratio) of *fleN* mutants and kanamycin-resistant 2P24 derivative (2P24-Km strain) that replaced the tetracycline resistance gene in the 2P24 ancestral strain with the kanamycin resistance gene. As a control, the 2P24 ancestral strain was co-inoculated with the 2P24-Km strain to regulate potential interference between the high-dose co-inoculated strains (48). *In vivo*, we soaked the germinating wheat seeds in the 10^7^ CFU/mL bacterial suspension, which was prepared by mixing the ancestral and mutant bacteria in equal volumes. Bacteria were harvested from the plant rhizosphere after 10 days, serially diluted, and respectively plated on LB medium with tetracycline and LB medium with kanamycin to count the cell number. *In vitro*, we inoculated 3 µL of 10^7^ CFU/mL mixed bacterial suspension to LB medium. After 40 h, we counted the cell number following the same procedure as *in vivo*. A relative competitive fitness was calculated as the ratio of *fleN* mutant/2P24-Km strain obtained from the plant rhizosphere (output) divided by the ratio in the inoculum (input). A minimum of six replicates were performed for each mutant. Cell densities were obtained during the bacterial competition assays for the tested clone and the kanamycin-resistant variant (2P24-Km strain) after 10 days of infection in plant rhizosphere (Table S4).

#### Individual colonization of *fleN* mutants measured in the wheat rhizosphere

To evaluate the individual colonization ability of the *fleN* mutants, germinating wheat seeds were soaked in a bacterial suspension containing the ancestral 2P24 strain or each of the four constructed *fleN* point mutant strains at a concentration of 10^7^ CFU/mL. The seeds were cultivated in a sterile substrate, and subsequently, bacterial communities were harvested from the wheat roots at days 2, 4, 6, 8, and 10, and serially diluted and plated on LB medium to count cell number, respectively. A minimum of six replicates were performed for each mutant.

#### Protein expression and purification

FleN wild-type and mutant proteins were cloned, overexpressed, and purified according to the method described previously and improved (49). FleQ protein was overexpressed and purified according to the method described previously and improved (28).

#### ATPase assay

The ATPase activity of wild-type and mutant proteins was measured using the ATPase Activity Assay Kit BC0960 (Solarbio). Briefly, the ATPase activity of FleN wt and mutants (5 µM) was carried out in a reaction buffer containing 20 mM HEPES (pH = 7), 2 mM MgCl_2_, 10 mM KCl, 1 mM dithiothreitol, and 250 mM NaCl. ATPase activity was determined by the amount of inorganic phosphorus produced by ATPase hydrolysis using the method of measuring absorbance at 660 nm. For the inhibition assay, the ATPase activity of 1 µM FleQ alone, as well as in the presence of FleN wt and mutants (5 µM), was carried out.

#### Bacterial two-hybrid system to detect FleN-FleQ interactions *in vivo*

We used the bacterial two-hybrid (BACTH [Bacterial Adenylate Cyclase-based Two-Hybrid) System Kit to detect and characterize FleN-FleQ protein interactions *in vivo* (EUROMEDEX).

#### RT-qPCR

Total bacterial RNA was isolated using TransZol Up Plus RNA Kit (TransGene) according to the manufacturer’s instructions. Complementary DNA (cDNA) was synthesized using the FastKing RT Kit (Tiangen). The mRNA levels of the *fleS* and *fleR* genes were measured by quantification of cDNA using SYBR Green (Accurate Biotechnology) and monitored using QuantStudio 1 Real-Time PCR System (Applied Biosystems). The *rpoD* gene was used as the reference gene (26). The primers used for qPCR are listed in Table S3.

### Statistical analysis

GraphPad Prism 8.0.2 was used to conduct the statistical analysis. All results were conducted at least three times in independent experiments. Two-tailed Student’s *t*-test was used to analyze two-group data. *P* < 0.05 was considered significantly different (*P < 0.05, **P < 0.05, ***P < 0.001, ****P < 0.0001; ns denotes no significance).

## Data Availability

The genome data are available under National Center for Biotechnology Information BioProject PRJNA1069306.

## SUPPLEMENTAL MATERIAL

Supplemental figures mbio.01023-25-s0001.docx Figures S1 to S10.

Table S1 mbio.01023-25-s0002.xlsx The cell densities of each evolved 2P24 population and the number of bacterial generations at each serial passage experiment.

Table S2 mbio.01023-25-s0003.xlsx Overview of the gene polymorphisms identified in all re-sequenced evolved populations of *Pseudomonas bijieensis* 2P24.

Table S3 mbio.01023-25-s0004.xlsx List of primers used in this study.

Table S4 mbio.01023-25-s0005.xlsx Cell densities obtained during the bacterial competition assays for the tested clone and the kanamycin-resistant variant (2P24-Km strain) after 10 days of infection in plant rhizosphere.

ASM does not own the copyrights to Supplemental Material that may be linked to, or accessed through, an article. The authors have granted ASM a non-exclusive, world-wide license to publish the Supplemental Material files. Please contact the corresponding author directly for reuse.
