# Supplementary material for: Experimental evolution of plant rhizobacteria reveals emerging adaptive mutations
Source: mBio. 2025 Jul 14;16(8):e01023-25. doi: 10.1128/mbio.01023-25 (PMC12345224; doi:10.1128/mbio.01023-25)
Supplement: Supplemental figures — Figures S1 to S10. [file mbio.01023-25-s0001.docx]

**Supplementary information**

Supplementary Figures 1–10, Supplementary Tables 1–4, Supplementary Results.


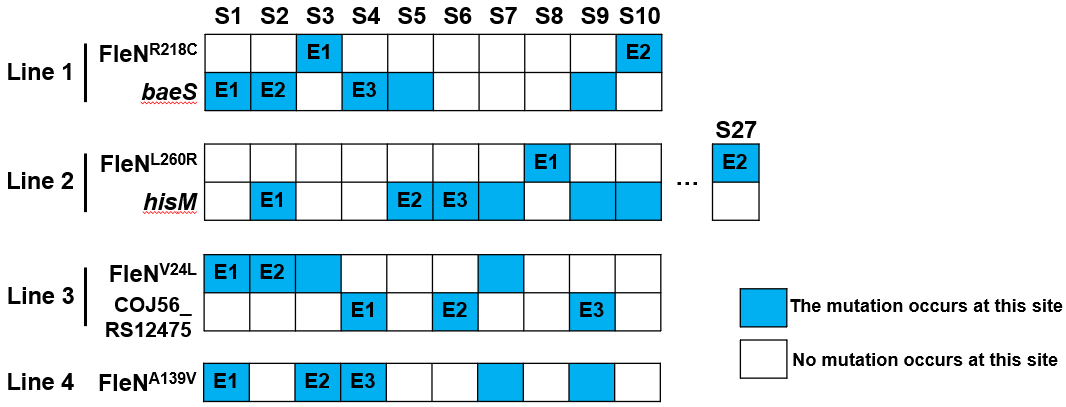


**Supplementary Fig S1** Validation of mutation sites by PCR and Sanger sequencing in 10 randomly stored strains (S1-S10) from each of the four final evolved populations. The blue/white color blocks represent occurrence/absence of mutations at that site, respectively. E1, E2 and E3 represent strains used for other phenotype determination experiments.


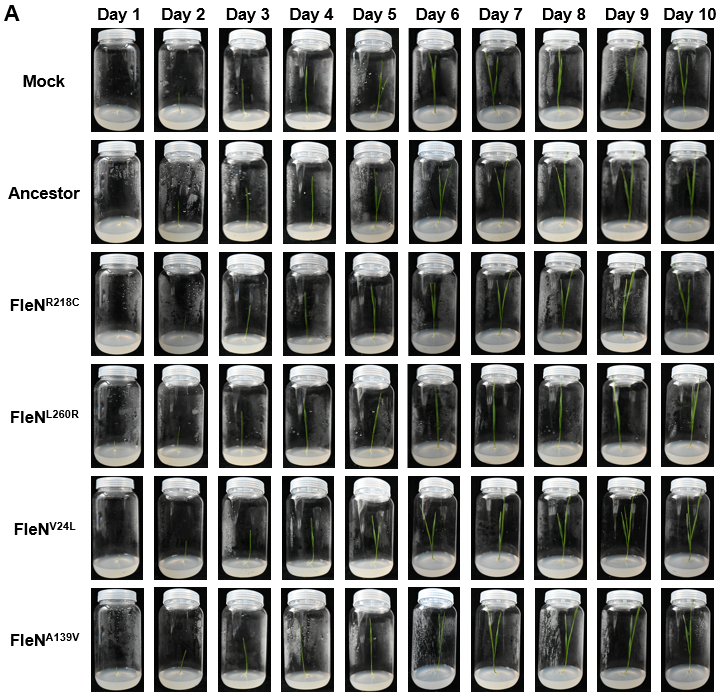


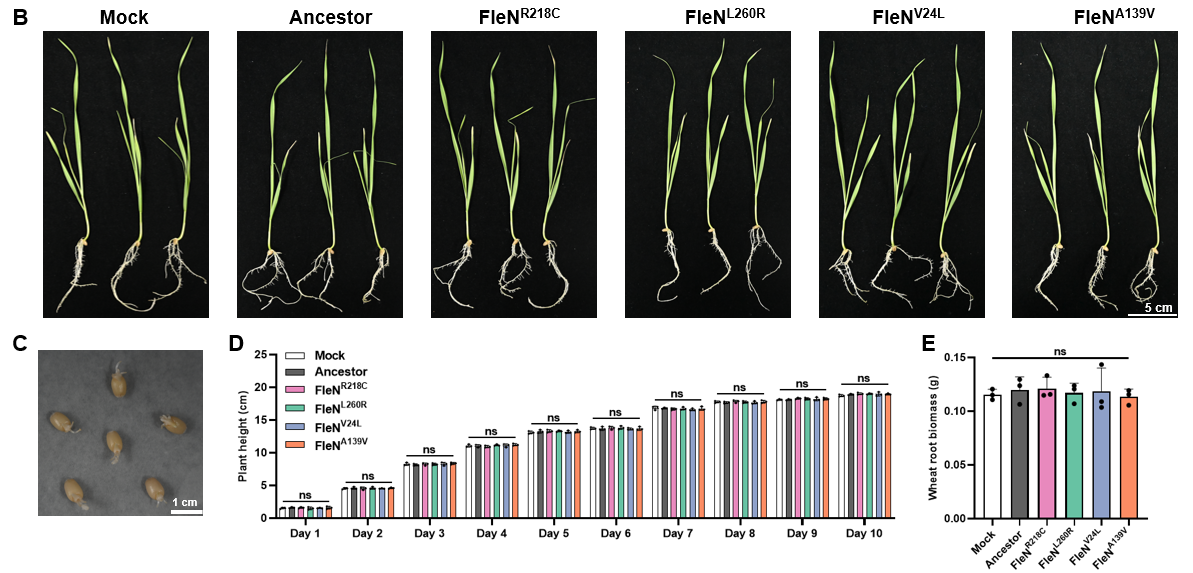


**Supplementary Fig S2** The *P. bijieensis* 2P24 ancestral strain and its *fleN* mutant strains have no effect on wheat growth. Growth phenotype (**A**) and statistical chart of wheat plant height (**D**) during the 10 days growth process after treating sterile germinating seeds (**C**) with ~10^7^ CFU/ml 2P24 solution and sterile water in a sterile culture system. Wheat morphology (**B**) and statistical chart of wheat root biomass (**E**) on the tenth day after the sterile germinating wheat seeds (**C**) were treated with ~10^7^ CFU/ml 2P24 solution and sterile water in the sterile culture system. (**C**) Morphology of wheat seeds after 36-hour germination under dark conditions on moist filter paper, following surface sterilization with 5% sodium hypochlorite solution for 30 minutes. Mean values of three replicates are given, and error bars indicate standard error in (**D**) and (**E**). Different colors represent different mutants in (**D**) and (**E**). Statistical significance of all the bar charts (*P* value) were calculated using two-tailed student’s *t* test. SD is calculated from three independent experiments in (**D**) and (**E**).


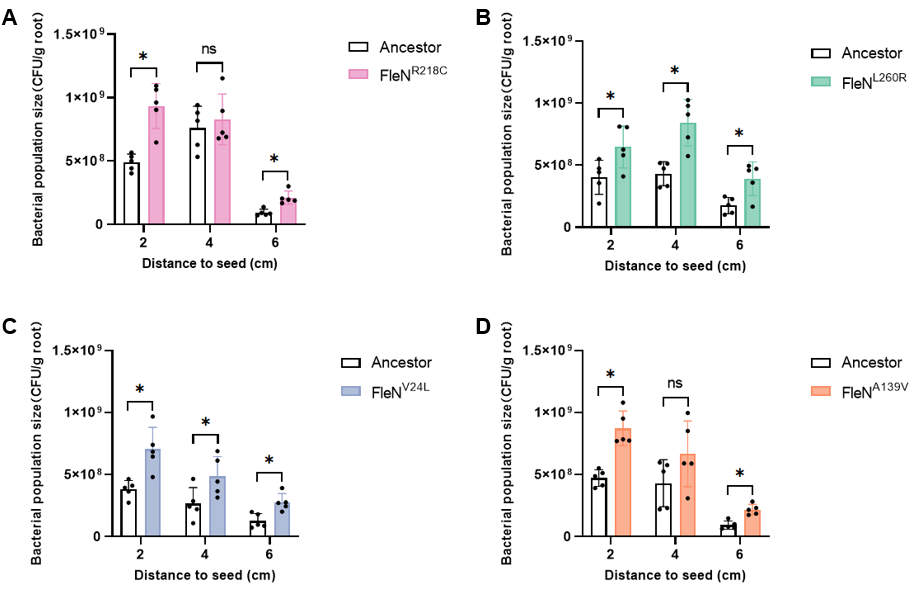


**Supplementary Fig S3** The FleN-SNP mutants exhibit stronger colonization ability across the root distances compared to the ancestral strain. The bar chart represents the colonization levels of the constructed FleN mutant clones (**A, B, C** and **D**) and the control ancestral 2P24 clone at different distances to the seed (2cm, 4cm and 6cm) in the wheat roots 10 days after co-inoculated (1:1). The y axis shows the bacterial population size in CFU/g root. Mean values of three replicates are given, and error bars indicate standard error. The statistical significance (*P* value) was calculated by two-tailed student’s t-test (**P* < 0.05).


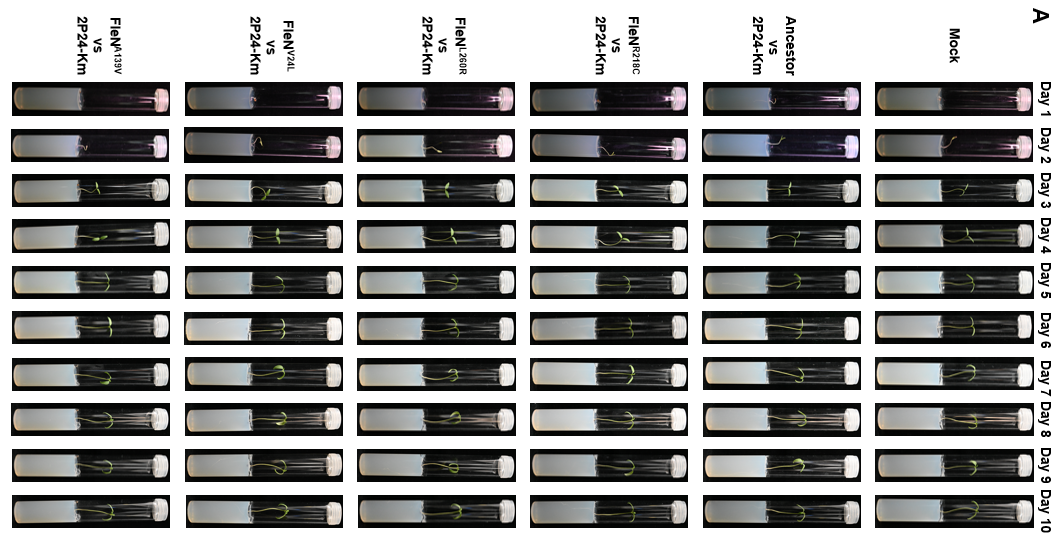


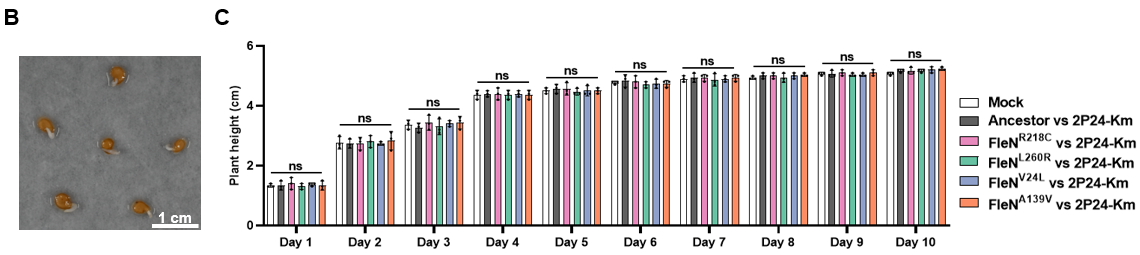


**Supplementary Fig S4** Growth phenotype (**A**) and statistical chart of tomato plant (**C**) height during the 10 days bacterial competitive colonization assay. (**B**) Morphology of tomato seeds after 72-hour germination under dark conditions on moist filter paper, following surface sterilization with 2.5% sodium hypochlorite solution for 5 minutes. Mean values of three replicates are given, and error bars indicate standard error in (**C**). Different colors represent different treatments in (**C**). Statistical significance of all the bar charts (*P* value) were calculated using two-tailed student’s *t* test. SD is calculated from three independent experiments in (**C**).


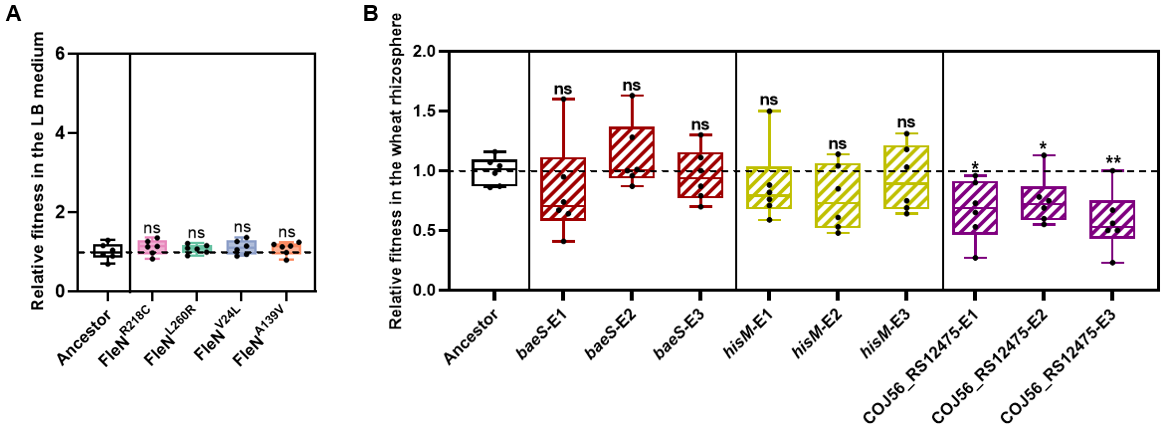


**Supplementary Fig S5** Competitive colonization ability of mutant strains and ancestral strains in different environment. (**A**) Box plot of the relative fitness of the control ancestral 2P24 clone and the constructed FleN-SNP mutant clones in the LB medium. (**B**) Box plot of the relative fitness of the control ancestral 2P24 clone and the other evolved mutant clones (*baeS*-E1, E2 and E3; *hisM*-E1, E2 and E3; COJ56_RS12475-E1, E2 and E3) in the wheat rhizosphere. All the strains were competed by 2P24-Km (2P24 carrying a kanamycin resistance cassette). The relative fitness was calculated based on the deviation from the initial 1:1 genotype ratio (dashed line) after direct competition. A minimum of six replicates were performed for each clone. Fitness values above the dashed line indicate a higher competitive advantage of mutants relative to their ancestral genotypes without mutations, whereas values below the dashed line denote for decreased competitive ability of mutants. Solid box: constructed mutant strains. Dotted box: evolved mutant strains. Different colors represent different evolutionary lines. Statistical analyses were performed using student’ s t-test. **P* < 0.05, ***P*<0.01.


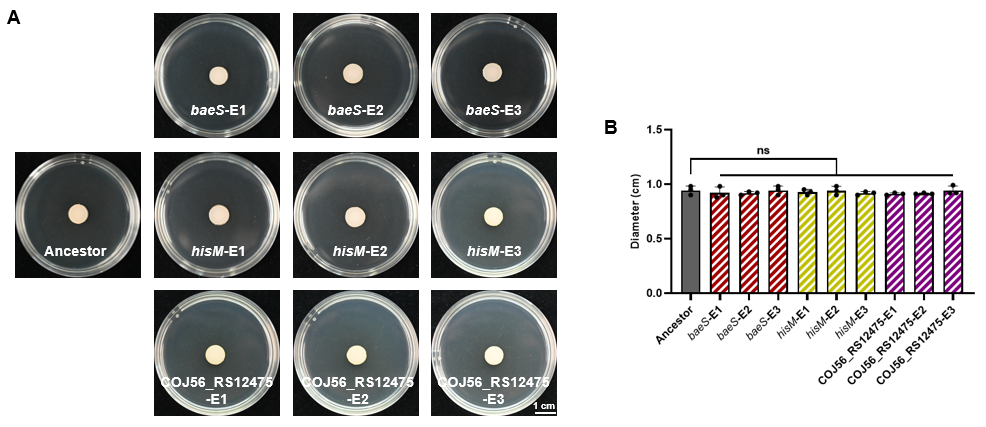


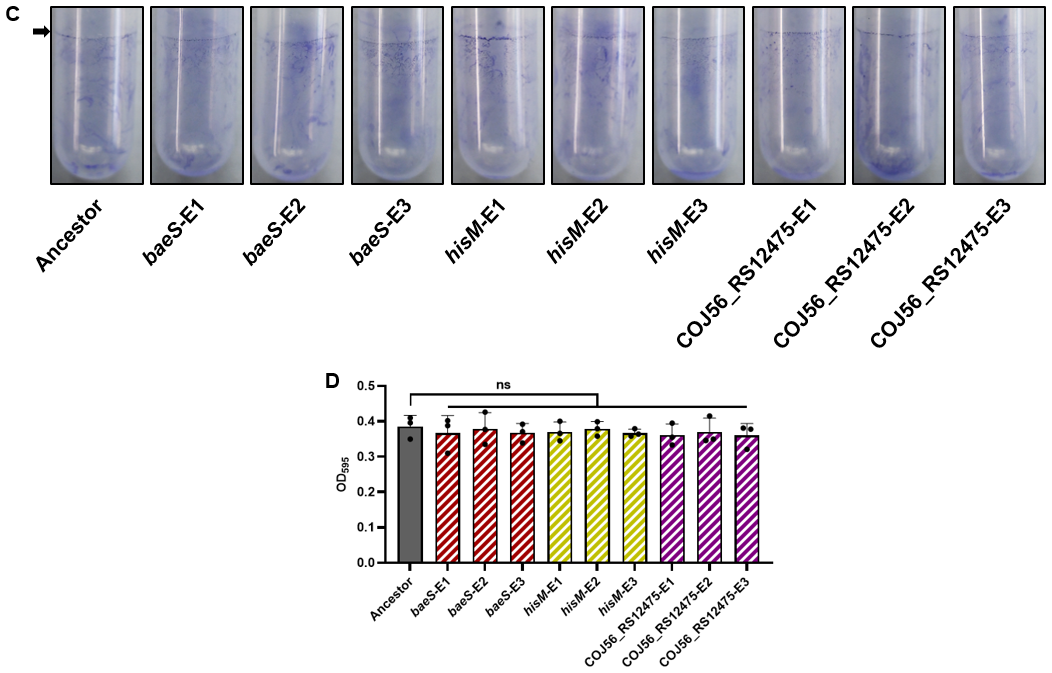


**Supplementary Fig S6** The determination of bacterial colonization-related phenotypes of the *P. bijieensis* 2P24 and its unique mutants from different Lines. (**A**)The swarming motility of the 2P24 ancestral strain (Ancestor) and the evolved unique mutant strains (Line 1: *baeS*-E1, E2 and E3; Line 2: *hisM*-E1, E2 and E3; Line 3: COJ56_RS12475-E1, E2 and E3) is shown on LB medium with 0.45% soft agar. (**B**) Bar chart represents the swarming zone for different strains of 2P24 on LB medium with 0.45% soft agar. The swarming zone was calculated by measuring the diameters of the colonies in (**B**). (**C**) The biofilm formation of the 2P24 ancestral strain (Ancestor) and the evolved unique mutant strains (Line 1: *baeS*-E1, E2 and E3; Line 2: *hisM*-E1, E2 and E3; Line 3: COJ56_RS12475-E1, E2 and E3) is shown. (**D**) Spectrophotometric quantification of biofilms formed by 2P24 ancestral strain and its unique mutants. Visual representation of biofilm formation by 2P24 ancestral strain and its unique mutants on tubes is shown. Biofilms were stained with Crystal Violet and determined at 595 nm after 20 hours of growth using a spectrophotometer. Different colors represent different evolutionary lines in (**B**) and (**D**). Statistical significance of all the bar charts (*P* value) were calculated using unpaired student’s *t* test. SD is calculated from three independent experiments in (**B**) and (**D**).


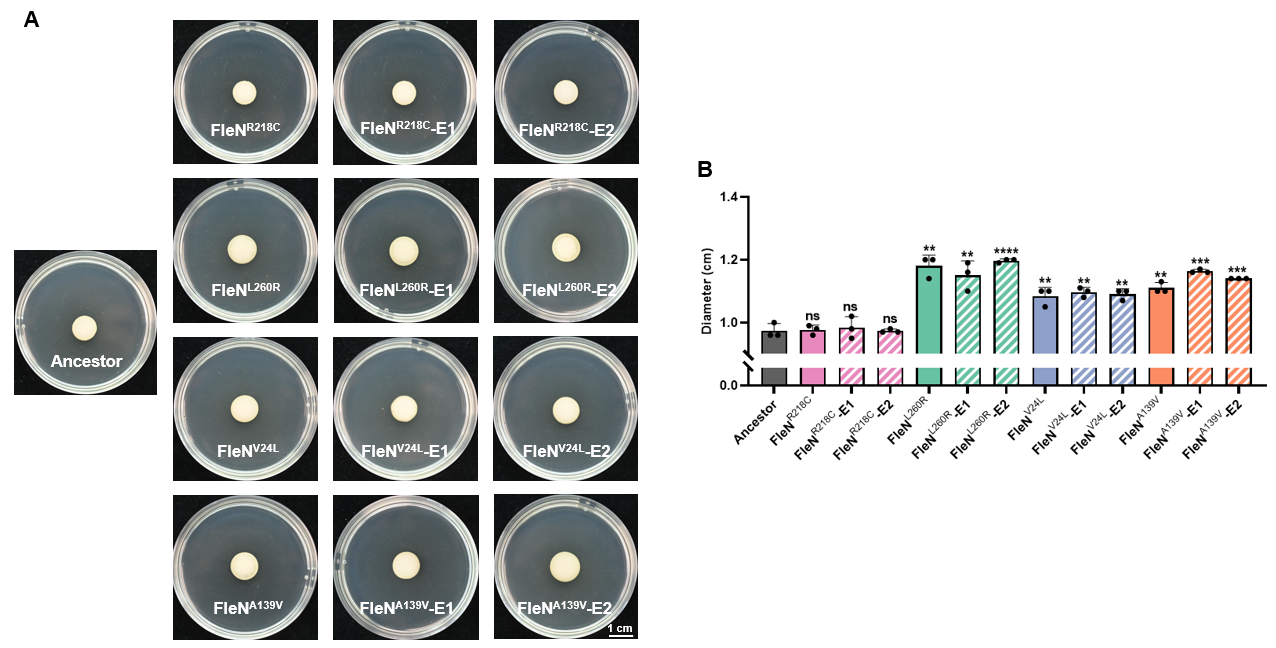


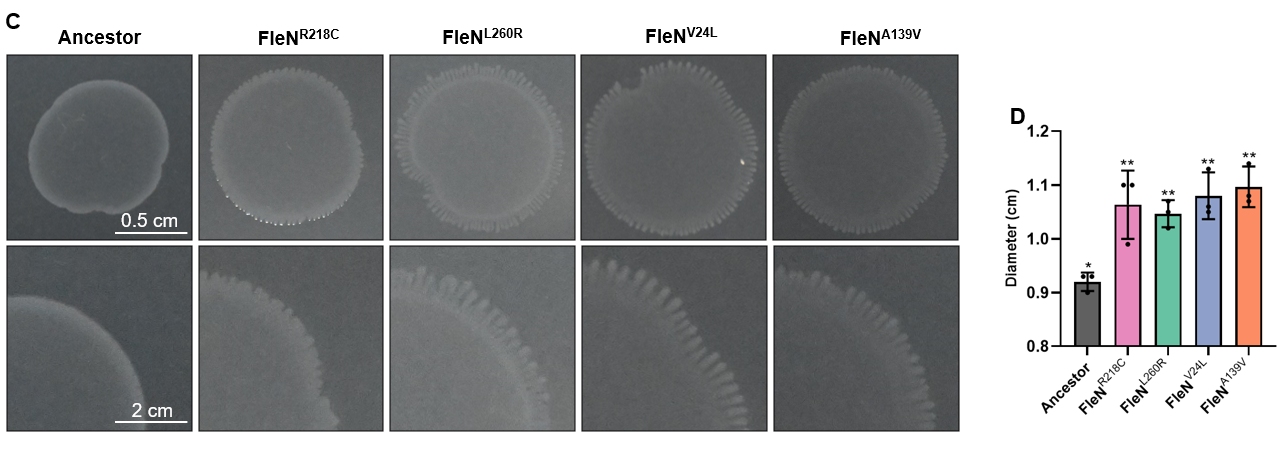


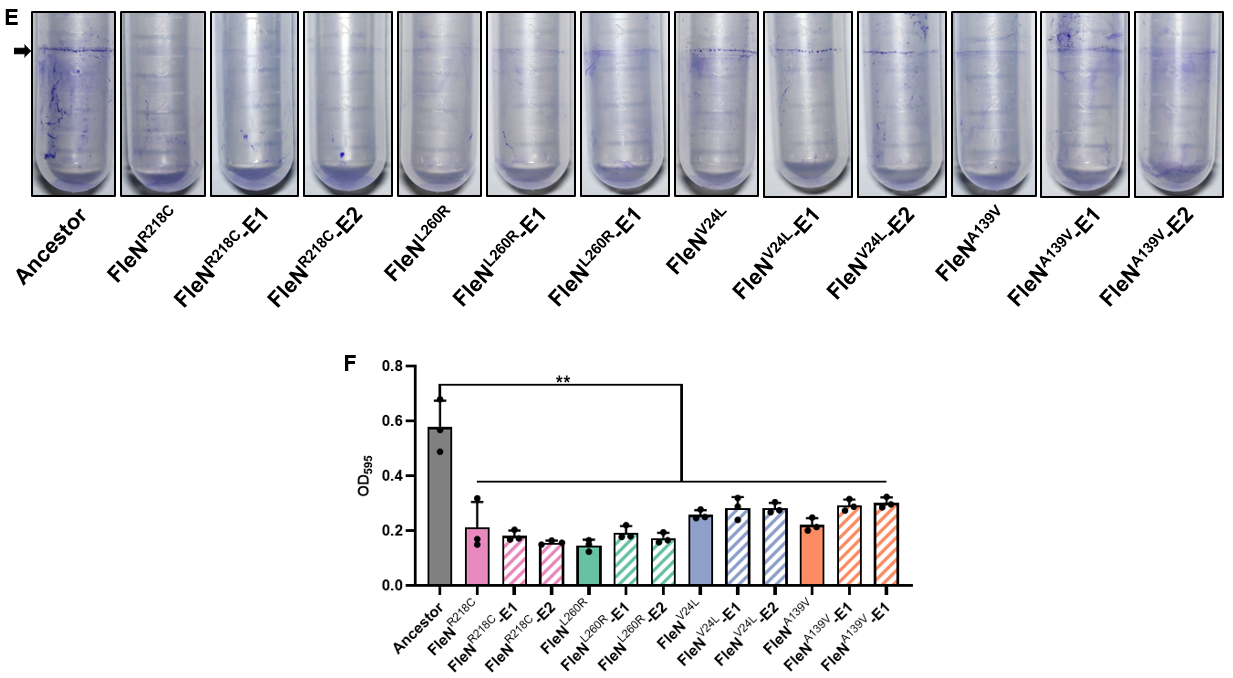


**Supplementary Fig S7** The determination of flagellar associated traits of the *P. bijieensis* 2P24 and its *fleN* mutants. (**A**) The swarming motility of the 2P24 ancestral strain (Ancestor), the constructed *fleN* mutant strains (FleN^R218C^, FleN^L260R^, FleN^V24L^ and FleN^A139V^) and the evolved *fleN* mutant strains (FleN^R218C^-E1, E2; FleN^L260R^-E1, E2; FleN^V24L^-E1, E2 and FleN^A139V^-E1, E2) is shown on LB medium with 0.45% soft agar. (**B**) Bar chart represents the swarming zone for different strains of 2P24 on LB medium with 0.45% soft agar. (**C**) The swarming motility of the 2P24 ancestral strain (Ancestor), the knockout strain (Δ*fleN*), the constructed *fleN* mutant strains (FleN^R218C^, FleN^L260R^, FleN^V24L^ and FleN^A139V^) is shown on casamino acid medium with 0.45% soft agar. (**D**) Bar chart represents the swarming zone for different strains of 2P24 on casamino acid medium with 0.45% soft agar. The swarming zone was calculated by measuring the diameters of the colonies in (**B**) and (**D**). The y axis shows the diameter of the swarming zone in centimeters in (**B**) and (**D**). (**E**) The biofilm formation of the 2P24 ancestral strain (Ancestor), the constructed *fleN* mutant strains (FleN^R218C^, FleN^L260R^, FleN^V24L^ and FleN^A139V^) and the evolved *fleN* mutant strains (FleN^R218C^-E1, E2; FleN^L260R^-E1, E2; FleN^V24L^-E1, E2 and FleN^A139V^-E1, E2) is shown. (**F**) Spectrophotometric quantification of biofilms formed by 2P24 ancestral strain and its *fleN* mutants. Visual representation of biofilm formation by 2P24 ancestral strain and its *fleN* mutants on tubes is shown. Biofilms were stained with Crystal Violet and determined at 595 nm after 20 hours of growth using a spectrophotometer. Mean values of three replicates are given, and error bars indicate standard error in (**B**)**,** (**D**) and (**F**). Different colors represent different evolutionary lines in (**B**)**,** (**D**) and (**F**). Statistical significance of all the bar charts (*P* value) were calculated using unpaired student’s *t* test. SD is calculated from three independent experiments in (**B**)**,** (**D**) and (**F**)*.* **P* < 0.05, ***P*<0.01, ****P*<0.001 and *****P*<0.0001.


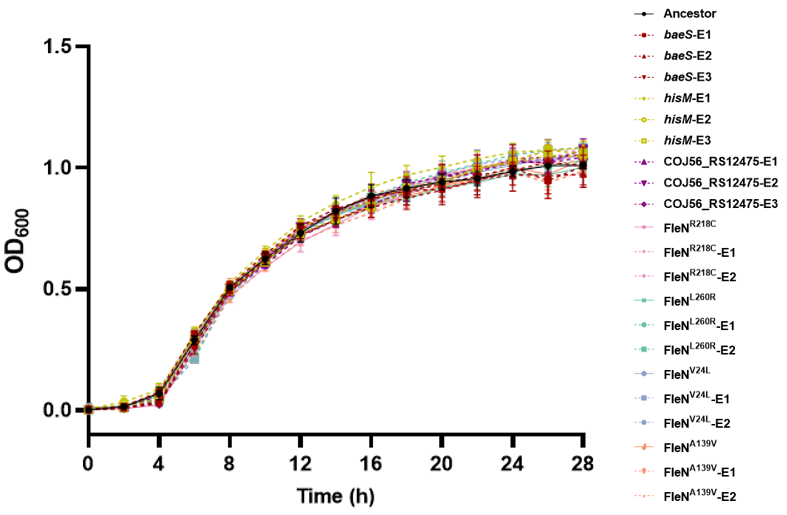


**Supplementary Fig S8** Growth of *P. bijieensis* 2P24 ancestral strain and its mutants in wheat root exudates. Bacterial yield was determined as the maximum optical density at 600 nm per two hours of growth using a spectrophotometer. Mean values of three replicates are given, and error bars indicate standard error.


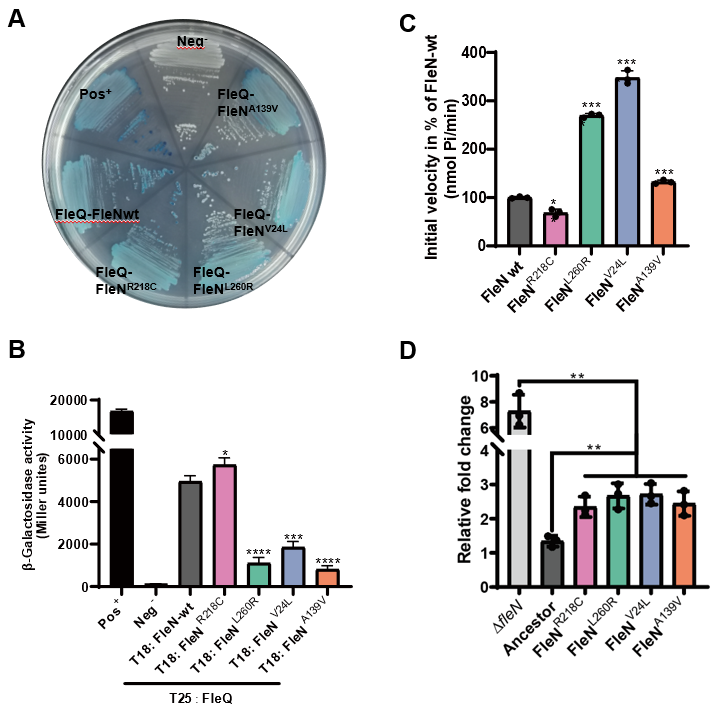


**Supplementary Fig S9** (**A**) and (**B**) The interactions of FleN-wt and its variants with FleQ *in vivo* was measured by bacterial two-hybrid to detect β-galactosidase activity. From light to deep blue, a Interaction strength from low to high is shown. (**C**) The ATPase activity of FleN wt and its variants (5 μM) was measured in the presence of 2 mM ATP. The initial velocities of ATP hydrolysis are plotted in percentage of FleN WT on the y axis (nmol Pi/min). (**D**) Relative transcript levels of *fleR* gene in various strains of *Pseudomonas bijieensis* 2P24 compared to the ancestor. Statistical significance of all the bar charts (*P* value) were calculated using unpaired *t* test. SD is calculated from three independent experiments in all cases. **P* < 0.05, ***P*<0.01, ****P*<0.001 and *****P*<0.0001.


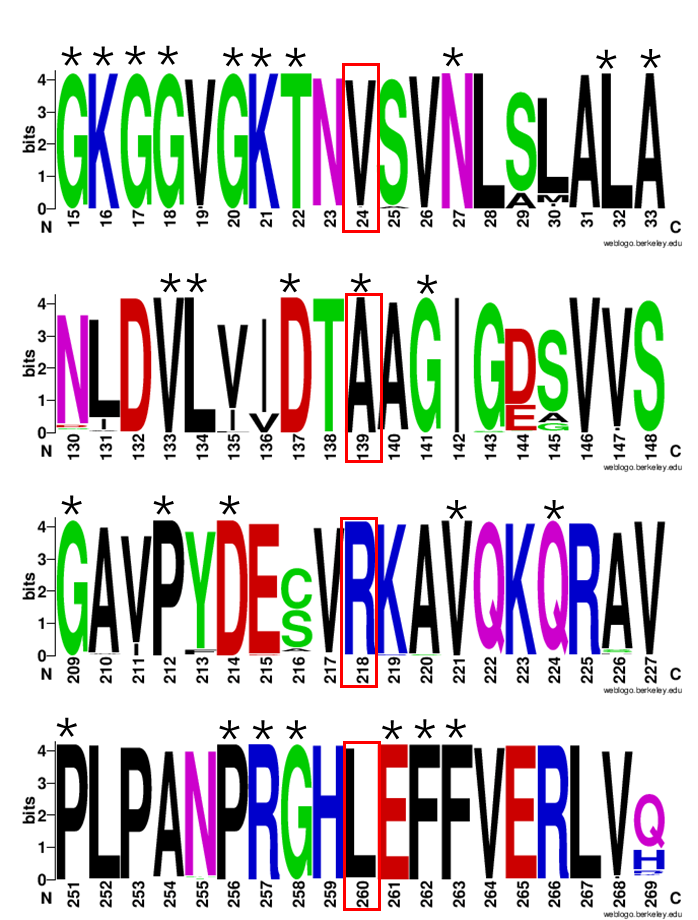


**Supplementary Fig S10** Multiple sequence alignment. The portion of the sequence alignment showing the region around evolved residue V24, A139, R218 and L260 (in red box) of FleN in the *P. bijieensis* 2P24 and 192 model strains of *Pseudomonas* genus. The residues marked with an asterisk indicate absolute conservation. Sequence LOGO created with weblogo for the giant motif (https://weblogo.berkeley.edu/logo.cgi).
